# Roles of the Non-Structural Proteins of Influenza A Virus

**DOI:** 10.3390/pathogens9100812

**Published:** 2020-10-03

**Authors:** Wenzhuo Hao, Lingyan Wang, Shitao Li

**Affiliations:** Department of Microbiology and Immunology, Tulane University, New Orleans, LA 70118, USA; whao2@tulane.edu (W.H.); lwang32@tulane.edu (L.W.)

**Keywords:** influenza, virulence, non-structural protein, NS1, PA-X, PB1-F2, innate immunity

## Abstract

Influenza A virus (IAV) is a segmented, negative single-stranded RNA virus that causes seasonal epidemics and has a potential for pandemics. Several viral proteins are not packed in the IAV viral particle and only expressed in the infected host cells. These proteins are named non-structural proteins (NSPs), including NS1, PB1-F2 and PA-X. They play a versatile role in the viral life cycle by modulating viral replication and transcription. More importantly, they also play a critical role in the evasion of the surveillance of host defense and viral pathogenicity by inducing apoptosis, perturbing innate immunity, and exacerbating inflammation. Here, we review the recent advances of these NSPs and how the new findings deepen our understanding of IAV–host interactions and viral pathogenesis.

## 1. Introduction

Influenza A virus (IAV) is a highly transmissible respiratory pathogen with the ability to cause seasonal epidemics and occasional pandemics, posing a considerable threat to global health [1,2,3,4]. IAV belongs to the Orthomyxoviridae family, featuring a segmented genome [5]. IAV has eight single-stranded, negative-sense viral RNA segments, which encode at least 10 proteins, including hemagglutinin (HA), neuraminidase (NA), matrix proteins 1 (M1) and 2 (M2), polymerase acidic (PA), polymerase basic 1 (PB1), polymerase basic 2 (PB2), nucleoprotein (NP), non-structural proteins 1 (NS1) and 2 (NS2). The IAV particle is enveloped with two abundant membrane proteins, HA and NA, and a few copies of the M2 ion channel protein. The M1 proteins lie beneath the lipid membrane and provide structure support to viral particles. Inside the viral particle are eight viral genome segments. Each RNA segment is coated with multiple copies of NP and associates with the trimetric RNA-dependent RNA polymerase complexes, consisting of PA, PB1 and PB2, to form viral ribonucleoprotein complexes (vRNPs).

IAV infection starts with the binding of HA to the sialic acid of host cell membrane receptors. The binding triggers receptor-mediated endocytosis and ferries the virus to the late endosome. In the late endosome, the low pH environment triggers the conformational change of HA to expose the fusion peptide that mediates the fusion between viral envelop and endosomal membrane. The ion channel M2 acidifies the viral particle, leading to the uncoating of vRNPs. Subsequently, vRNPs are released from endosome into cytoplasm through the fusion pore. Released vRNPs translocate into the nucleus, where the negative-sense viral RNA functions as a template for generating viral mRNA and complementary RNA. Viral mRNAs are exported to the cytoplasm for translation. Complementary RNA serves as a template for the synthesis of more viral RNA. Progeny vRNPs are exported to the cytoplasm assisted by M1 and NS2. Under the plasma membrane, the vRNPs, together with HA, NA, M1 and M2, are packaged as viral particles. Finally, the budding occurs, and new virions are released from the host cell by NA-mediated cleavage of the sialic acid.

**Figure 1:**
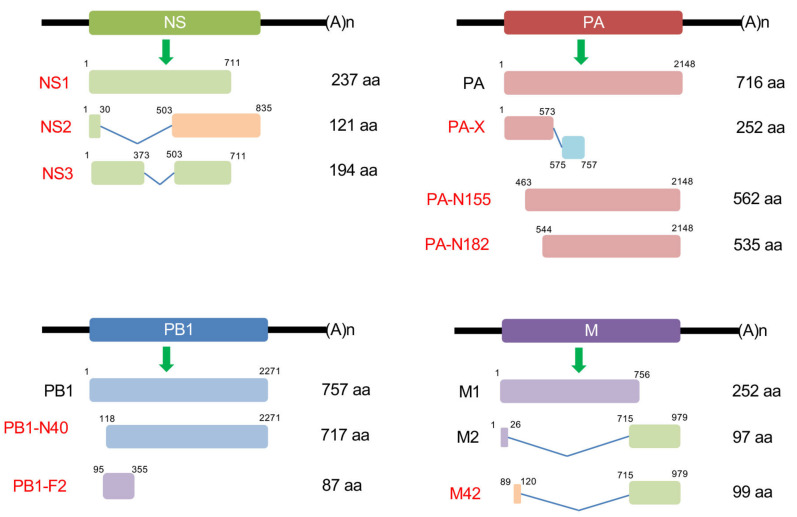
Schematics of the NS, PA, PB1 and M genome segment of influenza A virus (IAV) and the transcription of non-structural proteins (NSPs). The expression and size of NSPs may vary in different IAV strains.

**Figure 2:**
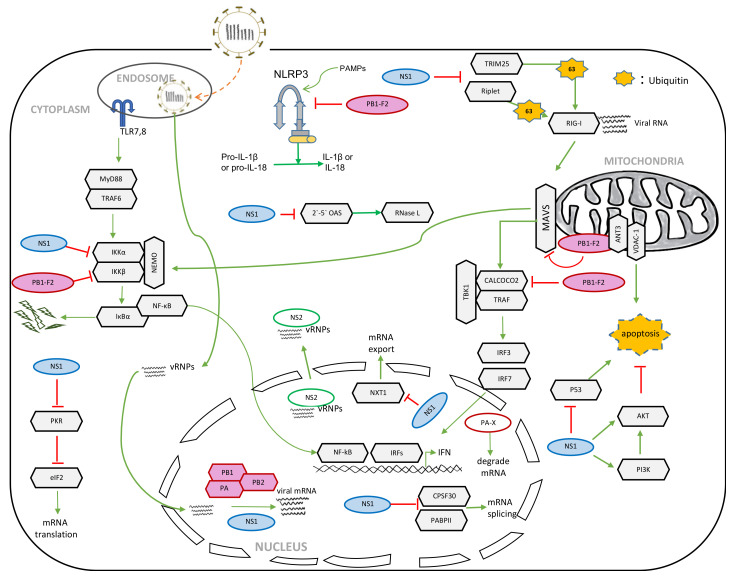
Roles of NSPs in IAV replication and innate host defense.

IAV is a plethora of zoonotic viruses that infect many other species, including humans, birds, and pigs. The host adaptation varies in different subtypes of IAV, and is largely determined by genetic and antigenic differences in the two glycoproteins, HA and NA. Currently, there are 18 HA subtypes and 11 NA subtypes that have been found in the wild [6]. All IAV strains express the eight structural proteins that are essential for IAV infection, replication and budding, as discussed above. IAV also expresses many non-structural proteins (NSPs), such as NS1, which are not packed in the IAV viral particle, but only expressed in the infected host cells. Recently, many NSPs have been discovered, including PA-X [7], PB1 frame 2 (PB1-F2), PB1-N40 [8], PA-N155 [9], PA-N182 [9], M42 [10] and NS3 [11]. Most of them are generated from the splicing, frame shift and truncation of the coding region of structural proteins (Figure 1). Although they are not essential (except NS2), NSPs have emerged as having crucial roles in host defense suppression, virulence and pathogenicity (Figure 2). Here we review the recent advances in the NSPs, and discuss how the new findings help us understand IAV pathogenesis. 

## 2. NS1

NS1 is a well-studied NSP with a molecular weight of approximately 26 kDa. NS1 has three distinct domains, an N-terminal RNA-binding domain (RBD) and an effector domain (ED) in the middle, followed by a C-terminal tail. Each domain interacts with distinct cellular factors and contributes to the multifunctional traits of NS1 [1,2,3,4,7]. NS1 is known to inhibit host innate immune defenses, limit host cell mRNA polyadenylation, engage with various cellular signaling pathways, and control viral RNA synthesis and splicing. We discuss the role of NS1 in each aspect below in detail.

### 2.1. Role of NS1 in the Suppression of Host Innate Immunity

Like other pathogens, IAV infection triggers host innate immune responses, the frontline of host defense. The innate immune system uses pattern recognition receptors (PRRs) in different cellular compartments to discriminate among microbial components that mark invading viruses. Many of these PRRs have been characterized, including the toll-like receptor (TLR) and retinoic-acid inducible gene I (RIG-I)-like receptors (RLRs) [12,13]. The RIG-I recognizes the double-stranded RNA (dsRNA) or 5’ triphosphate RNA generated by the viral replication of RNA viruses in the cytoplasm [14,15,16,17,18,19]. The engagement of viral RNA induces the conformational change of RIG-I and several post-translational modifications, such as the K63-linked ubiquitination and oligomerization of RIG-I [20,21,22]. The oligomerized RIG-I binds the mitochondrial antiviral signaling protein (MAVS), which leads to the oligomerization of MAVS and the recruitment of TANK-binding kinase 1 (TBK1) and IKKα/β. Subsequently, TBK1 phosphorylates interferon regulatory factors (IRFs), which results in the nuclear translocation of IRFs. At the same time, IKK mediates the phosphorylation and degradation of IκB, leading to the release of the cytosolic sequestered NF-κB. IRFs and NF-κB form active transcriptional complexes to activate type I interferon (IFN) expression (Figure 2).

The critical role of NS1 in the suppression of host innate immunity was first evidenced by the NS1 deletion of IAV that potently induced the production of IFN in vivo [23]. One mechanism involves NS1 inhibiting IRF3 activation by blocking RIG-I signaling [24]. As discussed above, K63-linked ubiquitination is critical for RIG-I activation, which is mediated by two E3 ligases, TRIM25 and Riplet [20,21]. It has been shown that NS1 interacts with TRIM25 and Riplet, and the sequestration of TRIM25 or Riplet inhibits RIG-I ubiquitination [25,26]. Furthermore, the NS1-mediated suppression of RIG-I is strain-dependent. NS1 proteins with an E at position 196 block the activation of IRF3, but NS1 proteins with K196 have little effect on IRF3 activation [27]. NS1 also impairs the NF-κB signaling pathway, a signaling branch from MAVS, by interacting with IKKα/β [28]. Additionally, NS1 binds to viral RNA through its RNA-binding domain, which blocks the activation of the 2′-5′ oligo (A) synthetase (OAS) /RNase L pathway by protecting viral RNA from the recognition of the innate immune sensor, 2′-5′ OAS [29].

### 2.2. Role of NS1 in the Shutoff of Host Gene Expression

In addition to perturbation of the innate immune signaling pathway, NS1 blocks the host defense by shutting off host global mRNA translation, including the expression of type I interferon genes and interferon-stimulated genes (ISGs). There are several proposed mechanisms. First, NS1 interacts with the 30 kDa subunit of cleavage and polyadenylation specificity factor (CPSF30) and prevents CPSF30 from binding to cellular pre-mRNAs, leading to the accumulation of unprocessed cellular pre-mRNAs in the nucleus and the inhibition of mRNA translation in the cytoplasm [30,31,32]. Several residues of NS1 are required for the interaction with CPSF30, including F103 and M106 [33]. F103 and M106 are conserved among most human IAVs, but are absent in some strains, such as PR8 and 2009 H1N1pdm [33,34]. Consistently, the NS1 of PR8 is unable to interact with CPSF30 [35,36]. Furthermore, L103F and I106M mutations in the NS1 of H5N1 viruses increase NS1 interaction with CPSF30 [35]. These replacements enhance viral replication and cause severe pathogenicity [35,37].

Secondly, NS1 also targets the poly(A)-binding protein II (PABII) of the cellular 3’-end processing machinery to shut off host protein expression [38], but the mechanism is different from the targeting of CPSF30. By inhibiting the ability of PABII to stimulate the processive synthesis of long poly(A) tails, NS1 causes the nuclear accumulation of cellular pre-mRNAs with short poly(A) tails [38]. In addition, NS1 further blocks the nuclear-cytoplasmic shuttling of PABII, which is critical for mRNA nuclear export [38]. Interestingly, both CPSF30 and PABII bind the effector domain of NS1, suggesting that CPSF30 and PABII might form a ternary complex with NS1 [38]. 

Thirdly, NS1 forms an inhibitory complex with NXF1/TAP, p15/NXT, Rae1/mrnp41 and E1B-AP5, which are the core constituents of the mRNA export machinery that directs mRNAs through the nuclear pore complex [39]. These interactions lead to the blocking of the nuclear export of fully processed host mRNAs. 

Lastly, the NS1 proteins of H3N2 viruses possess a histone-like sequence (histone mimic), i.e., the sequence ARSK (positions 226–229) at their C-terminus that is analogous to the first four amino acids (ARTK) of histone H3 [40]. Similar to the histone H3K4 methylation, the K in the NS1 ARTK sequence is also methylated. The methylated NS1 sequence acts as a ‘histone mimic’ that binds to the human PAF1 transcription elongation complex (hPAF1C). The binding of NS1 to hPAF1C depends on the NS1 histone mimic, resulting in the suppression of the hPAF1C-mediated transcriptional elongation of host genes, including antiviral genes [40]. 

As discussed above, NS1 shuts off host gene expression by the suppression of transcription, interference of pre-mRNA maturation, and the inhibition of mRNA nuclear export. All these events happen in the nucleus, suggesting that IAV inhibits host gene expression prior to mRNA translation in the cytoplasm. It is a smart viral strategy because the virus must utilize the host translation system for viral protein synthesis. Furthermore, the cytosolic NS1 binds the cellular double-stranded RNA sensor, protein kinase R (PKR), to prevent the translation shutoff [41,42,43]. PKR is activated by dsRNA or by its activator, the protein activator of the interferon-induced protein kinase (PACT), resulting in the phosphorylation of target proteins, including the α subunit of the eIF2 translation initiation factor (eIF2α). Phosphorylation of eIF2α leads to the inhibition of global protein synthesis in infected cells, thereby inhibiting virus replication. NS1 is also found to interact with PACT [44] and a PKR substrate, NF90 [45]. Whether these interactions alone are sufficient to block PKR activity is not clear. Overall, the nuclear and cytoplasmic NS1 proteins have distinct roles, and co-opt to ensure viral protein synthesis while shutting off host gene expression.

### 2.3. Role of NS1 in Apoptosis

NS1 activates the PI3K/Akt pathway to delay virus-induced apoptosis, thereby providing sufficient time for virus replication [46,47,48,49]. NS1 binds directly to the SH2 domains of the p85 beta regulatory isoform of PI3K [47,49,50,51,52,53]. Recent studies also found that NS1 activates PI3K by binding the N-terminal SH3 domains of Crk and/or CrkL [48,54,55]. However, the effect of NS1 on the PI3K pathway is dependent on viral strain, cell type, time course, and the subcellular localization of the activation events [56]. NS1 also directly interacts with and activates Akt activity [57]. Additionally, NS1 interacts with p53 and inhibits p53-mediated transcriptional activity and apoptosis, thereby promoting cell survival [58]. By contrast, NS1 facilitates apoptosis through interactions with heat shock protein 90 [59] and alpha tubulin [60]. 

### 2.4. Role of NS1 in Viral Replication 

NS1 proteins exploit host factors to facilitate viral replication, RNA splicing, and viral particle formation. For example, NS1 interacts with translation initiation factor 4GI (eIF4GI) and polyadenine binding protein 1 (PABP1) [61]. These cellular factors form a complex with NS1 in viral mRNA translation initiation complexes, and promote the translation of viral mRNA, but not cellular mRNA [61]. NS1 also regulates viral replication and gene expression through interactions with two RNA helicases, RNA helicase A (RHA) [62] and DExD-Box Helicase 21 (DDX21) [63]. RHA promotes viral polymerase activity and viral replication [62]. DDX21 restricts IAV by binding PB1, leading to the inhibition of polymerase assembly and a reduced synthesis of viral RNA and protein. NS1 overcomes this restriction by binding to DDX21 and displacing PB1 [62]. In the late stage of the IAV replication cycle, the NS1 proteins participate in the morphogenesis of the virus particles, although the mechanism is unknown [64].

### 2.5. Virulence Determinant in NS1

The ability of IAV to adapt to various hosts through reassortment events ensures a constant generation of a plethora of new strains with different degrees of virulence. IAV virulence is multigenic and determined by the constellation of genes within a particular strain in a specific host [1,65]. These virulence determinants contribute to virus pathogenicity, transmissibility and pandemic potential [1,65]. Several IAV virulence determinants have been identified in NS1, such as 92E [66], the C-terminal PDZ ligand motif [65,67,68,69], and the 103F and 106M sites [30,31,32,35]. The 103F and 106M sites are discussed above. The position 92 of NS1 is a glutamic acid, rather than aspartic acid in H5N1 HPAI viruses [66]. The 92E is critical for the virus to escape from host antiviral cytokine responses in swine [66]. Similarly, the P42S substitution in NS1 of H5N1 IAV results in an increased virulence in mice and a reduced level of IFN-α/β production in vitro [70]. The PDZ ligand motif in NS1 is the C-terminal motif, consisting of “ESEV” in avian viral isolates or “RSEV” in human viral isolates [65,67,68,69]. The PDZ binding motif of NS1 is cell type- and viral species-dependent [65,67,69]. When the PDZ ligand motif of either pandemic 1918 or H5N1 avian IAV was added to the NS1 of WSN IAV, the recombinant virus became more pathogenic [65]. Many PDZ domain-containing proteins have been reported to interact with NS1, including PDlim2 [71], Scribble [72,73], PSD-95 [74], Dlg1 [72,73], MAGI-1 [72,73], MAGI-2 [72,73] and MAGI-3 [72,73]. Although not all these proteins are well-studied, these PDZ proteins might contribute to diverse biological functions, such as cellular tight junction [75], nitric oxide production [74] and interferon expression [76].

## 3. NS2

In addition to NS1, segment 8 of the IAV genome encodes a second viral gene called NS2, derived from a spliced form of the segment mRNA transcript [77,78]. NS2 is highly conserved across all sequenced IAV strains. It comprises an N-terminal domain with two nuclear export signals (NESs) and an amphipathic C-terminal domain of two α-helices [79]. NS2 was thought of as an NSP when discovered; however, later studies found a small amount of NS2 inside the viral particle [80,81]. Furthermore, NS2 is an essential viral gene directly pertaining to the virus life cycle, as the disruption of the NES results in a complete loss of virus replication [82,83]. These features are absent in all other NPSs discussed in this paper. However, due to the historical name of NS2, we include it in this review. 

### 3.1. Role of NS2 in vRNP Nuclear Export

NS2 is also known as nuclear export protein (NEP) due to its role in the nuclear export of vRNP complexes [79,83,84,85,86,87]. It has two NESs; one is located between residues 12 and 21 [83,87] while the other is located from residue 31 to 40 [88]. The NES in the N terminus of NS2 interacts with the chromosome region maintenance 1 (CRM1), whereas the C terminus binds the M1 protein, thereby forming the CRM1–NS2–M1–vRNP nuclear-export complex [79,81,84,87,88]. The complex mediates progeny vRNP export from the nucleus to the cytoplasm. Recently, several cellular factors have been reported to regulate NS2-mediated vRNP nuclear export. For example, the chromodomain helicase DNA binding protein 3 (CHD3) was found to interact with the NES of NS2, which facilitates vRNP export [89]. Inhibition of the mitogen-activated protein kinase (MAPK) signaling also perturbs NEP-mediated exportation [90]. 

After export to the cytoplasm, the vRNP further traffics to the plasma membrane for progeny virion assembly. Although NS2 has not yet been found to be associated with the endomembrane that is required for vRNP trafficking, it was reported that NS2 interacts with the plasma membrane-associated F1Fo ATPase [91]. The interaction of NS2 with F1Fo ATPase facilitates influenza virion formation/budding [91]. It will be interesting to know whether NS2 accompanies vRNP during trafficking to the cell membrane, or whether NS2 directs vRNP to the endomembrane system after nuclear export.

### 3.2. Role of NS2 in Viral RNA Transcription and Replication

Studies on the defective interfering particles shed the first light on the role of NS2 in viral transcription and replication. Mutation in NS2 was found to lead to the generation of defective interfering particles, but at the same time to cause aberrant replication [92,93]. Subsequently, NEP was demonstrated to stimulate viral RNA replication or accumulation [94]. Conversely, a study showed that NS2 downregulates viral RNA synthesis in a mini-replicon system [95]. This could be reconciled by the observation that a high expression of NS2 inhibited viral polymerase activity, but a low amount of NS2 promoted the vRNP activity [96]. The optimal expression of the NS2 of some avian H5N1 influenza viruses is required for efficient replication in mammalian cells, which plays a critical role in host adaptation [96]. A recent study further showed that the optimal expression of NS2 is essential in determining the levels of virus replication [97]. However, the mechanism by which NS2 regulates viral polymerase activity is not clear. Recently, it has been found that NS2 interacts with aminoacyl-tRNA synthetase (AIMP2) [98]. The interaction facilitates the switch from ubiquitination to SUMOylation of M1, thus increasing viral replication [98]. Several cellular factors are found to interact with NS2 by proteomics [36,99]. It will be interesting to know whether these interactions contribute to the role of NS2 in viral replication. More importantly, future work needs to determine whether these new interactions lead to a new function of NS2 in viral infection and pathogenesis.

## 4. PB1-F2

PB1-F2 is a small NSP, encoded by an alternate (+1) reading frame within the PB1 gene [100,101,102]. Unlike NS1 and NS2, the PB1-F2 protein is not in all IAV strains. It also has various lengths, amino acid sequences, and subcellular localizations in different strains, contributing to the strain-specific pathogenicity. PB1-F2 was first found to localize in the mitochondria and induce cell death [100]. Interestingly, the mitochondrial localization of PB1-F2 is strain-dependent [103]. Recent works further found that PB1-F2 blocks the RIG-I signaling pathway, modulates NLRP3 inflammasome activation, enhances viral polymerase activity, and promotes viral pathogenicity, as discussed below.

### 4.1. Role of PB1-F2 in Apoptosis

PB1-F2 proteins localized in the mitochondria from selected IAVs are known to be pro-apoptotic [100]. PB1-F2 proteins target the inner mitochondrial membrane via a C-terminal helix [104,105], and translocate into mitochondria through Tom40, the import channel of the mitochondrial outer membrane [106]. Interestingly, the synthetic PB1-F2 peptide creates pores in planar lipid membranes in vitro [107] and induces cell death via BAK/BAX-mediated cytochrome c release from mitochondria [108]. In cells, PB1-F2 interacts with two mitochondrial membrane proteins, voltage-dependent anion channel 1 (VDAC-1) and adenine nucleotide translocator 3 (ANT3) [101]. ANT3 and VDAC-1 are two major components of the permeability transition pore of the mitochondrial membrane, which are implicated in the mitochondrial permeability transition during apoptosis. The interaction between ANT3 and VDAC-1 causes the mitochondrial permeabilization and subsequent apoptosis. Conversely, the nucleotide-binding oligomerization domain-like receptor (NLRX1) interacts with PB1-F2 and prevents mitochondrial stress-induced apoptosis [109].

### 4.2. Role of PB1-F2 in Host Innate Immune Responses

The outer membrane of the mitochondria is the signaling platform for MAVS, the downstream effector of RIG-I. MAVS recruits the IKK family members, such as IKKα/β and TBK1, and consequently the activating transcription factors NF-κB and IRF. Recent studies show that the mitochondrial PB1-F2 suppresses viral RNA-elicited innate immunity by modulating MAVS in the mitochondria. PB1-F2 localizes to the mitochondria’s inner membrane space and causes the attenuation of membrane potential, which results in the suppression of RIG-I signaling [106]. PB1-F2 is also found to bind the transmembrane domain of MAVS and dissipates the mitochondrial membrane’s potential [110,111]. However, it is not clear whether there is a correlation between MAVS binding and membrane potential. Additionally, PB1-F2 interacts with IKKβ and inhibits NF-κB activation [112]. PB1-F2 also interacts with CALCOCO2, a TBK1-binding protein [113], and inhibits TRAF2/3-mediated IFN induction [114]. Although PB1-F2 subverts host IFN production signaling pathways, PB1-F2 modulates NF-κB signaling and promotes the expression of pro-inflammatory cytokines through TRAF6 [114]. The comparison of wild type and PB1-F2 deletion IAV found that PB1-F2 expression induces the expression of genes linked to cell death, inflammatory response, and neutrophil chemotaxis [115,116]. The aberrant activation of innate immunity, such as pro-inflammatory cytokine, is an indicator of severe infection with high pathogenic IAVs. Therefore, it will be of great interest to elucidate the pathogenic role of PB1-F2 in the aberrant innate immune responses and the underlying mechanisms in the future.

### 4.3. Role of PB1-F2 in Inflammasome Activation

Inflammasome is a large and highly ordered cytosolic complex that mediates the proteolytic processing of interleukin (IL)-1 family members, such as IL-1β and IL-18, to their mature active form [117]. NOD-like receptors (NLRs) are a family of intracellular sensors that recognize microbial products and activate the inflammasome [118]. As discussed in Section 4.2, a study showed that the mitochondria inner membrane localization of PB1-F2 causes the attenuation of membrane potential [106]. The study also showed that the reduction of membrane potential not only suppresses RIG-I signaling, but also blocks NLRP3 inflammasome activation by the co-transfection of inflammasome components in HEK293T cells. Another study further showed that the PB1-F2 of IAV H7N9 selectively suppresses dsRNA-induced NLRP3 inflammasome activation by inhibition of MAVS-NLRP3 interaction [119]. However, whether PB1-F1 inhibits the activation of inflammasome during viral infection is not clear. Conversely, mice infected with the PB1-F2 deletion mutant PR8 virus, ΔPB1-F2/X31, showed decreased IL-1β secretion within BAL fluid [120]. Furthermore, the intranasal administration of PB1-F2 C-terminal peptide to mice exacerbated inflammasome activation, resulting in severe pulmonary inflammation with excessive production of IL-1β and IL-18 [108,120,121]. NLRP3 deficiency and the specific inhibitor MCC950 ablated the PB1-F2 peptide-induced inflammasome activation [120,121], suggesting that PB1-F2 activates NLRP3 inflammasome. Theses striking observations raise a couple of questions: First, what causes the different outcomes between intracellular and extracellular PB1-F2? It is plausible that the overwhelming peptide causes mitochondrial stress, leading to inflammasome activation [122]. Secondly, PB1-F2 shows strain-specificity on NLRP3 inflammasome, but the determinant is unknown. As PB1-F2 is a short protein, future work on mutagenesis will help reveal the critical site(s). 

### 4.4. Role of Cytosolic and Nuclear PB1-F2

PB1-F2 has been reported to interact with cytosolic proteins, IKKβ [112] and CALCOCO2 [114], indicating a cytosolic localization of PB1-F2. As discussed above, these cytosolic interactions lead to the suppression of the NF-κB and IRF3 innate immune signaling pathways. In addition, the PB1-F2 of H5N1 IAV is also found in the nucleus [103]. The PB1-F2 of H5N1 IAV has little effect on apoptosis but enhances viral polymerase activity [103]. Another study showed that the PB1-F2 of PR8 IAV is also localized in the nucleus and interacts with PB1, resulting in an increased PB1 retention in the nucleus and enhanced polymerase activity [123]. However, many natural isolates of IAV, such as the 2009 H1N1pdm, completely lack a PB1-F2 open reading frame (ORF) due to multiple stop codons. Furthermore, recent studies found a minimal effect of PB1-F2 on viral replication in cultured cells and in mice [124,125], suggesting that PB1-F2 is not essential for viral polymerase activity. Nonetheless, the discovery of the cytosolic and nuclear localization will further the findings concerning new roles of PB1-F2 in viral pathogenesis.

### 4.5. Virulence Sites in PB1-F2

An N66S mutation in the PB1-F2 sequence is considered a virulence determinant. Highly pathogenic strains, including pandemic 1918 H1N1 and A/Hong Kong/156/1997(H5N1), carry the S66 to enhance pathogenicity in mice [102,126]. By contrast, nonpathogenic and less pathogenic IAVs have an N66 in PB1-F2 [102]. The virulence of S66 is characterized by early inhibition of IFN, increased levels of pro-inflammatory cytokines, and enhanced infiltration of immune cells in the lung [102,126]. The N66S mutation enhances the binding of PB1-F2 to MAVS [111], which at least partially explains the mechanism of S66 virulence. Moreover, the virulence of S66 is also dependent on host specificity. For example, the S66 in the PB1-F2 of A/Viet Nam/1203/2004(H5N1) increased viral virulence in mice, but not in birds [127]. Similarly, the S66 increased the virulence of A/swine/Kansas/77778/2007(H1N1) in mice, but not in pigs [128]. In addition, the rescue of 2009 H1N1pdm with a PB1-F2 with S66 has minimal effects on virulence in mice and ferrets, although the increased expression of pro-inflammatory cytokines is found [124]. 

Other than the S66, several sites (L62, R75, R79 and L82) have been found to be pro-inflammatory [129]. PB1-F2-derived peptides containing these pro-inflammatory sites cause significant morbidity, mortality and lung inflammation in mice [129]. These sites also contribute to superinfection with Gram-positive respiratory pathogens [130]. In addition, three other sites (I68, L69 and V70) are identified as a cytotoxic sequence that promotes PB1-F2’s cytotoxicity and contributes to the pathogenesis of primary viral and secondary bacterial infection [131]. These three sites, plus F71, increase the half-life of PB1-F2 and interferon antagonization [132].

Overall, PB1-F2 is an accessory viral protein that plays multiple roles in cell death, innate immunity, inflammasome and vRNP activity. PB1-F2 proteins demonstrate genetic and functional diversities, manifested by various lengths, amino acid sequences, cellular localizations and functions in different strains. These traits contribute to the strain-specific pathogenicity, which makes PB1-F2 flexible and adaptable in maintaining the optimal virulence and replication efficiency.

## 5. PA-X

PA-X is an accessory viral protein that is translated as a +1 frameshift ORF from the PA segment [7] (Figure 1). During translation, the ribosome shifts to a uracil (U)-rich stretch, followed by a rare codon in the PA mRNA [133]. The rare codon is decoded more slowly, thereby promoting ribosomal frameshifting. The one nucleotide frameshift results in the generation of PA-X that comprises the same first N-terminal 191 amino acids of PA protein and a unique short C-terminus extension containing 41 or 61 amino acids. The frameshift motif is highly conserved, and most IAV PA-X proteins have a 61 amino acid extension [134]. Of note, the PA-X of 2009 H1N1pdm only encodes a 41 amino acids extension [134].

### 5.1. Role of PA-X in the Shutoff of Host Gene Expression

Like NS1, PA-X is also known to shut off host translation, but through a different mechanism [7,135]. With the N-terminal endonucleolytic domain of PA, PA-X possesses endonucleolytic activity and the ability to degrade RNA [7,135]. Interestingly, PA-X selectively degrades host RNA polymerase II (Pol II)-transcribed mRNAs and non-coding RNAs, while it has no effect on the production of pol I and pol III, and viral mRNA [136,137]. What determines the selectivity is not very clear, and it may be dependent on cellular factors. A study found that the complete degradation of host mRNAs following PA-X-mediated endonucleolytic cleavage is also dependent on the cellular 5’->3’-exonuclease Xrn1 [136]. Moreover, a recent study further showed that PA-X usurps RNA splicing machinery to selectively target host nascent mRNA for destruction [138]. 

PA-X localizes in the cytoplasm and nucleus, and nuclear accumulation is associated with PA-X’s shutoff activity [136]. Several basic amino acids in the C-terminal region (amino acids 192–200) of PA-X are required for nuclear localization and host shutoff ability [136,139]. Furthermore, recent studies also found that the amino acids 233–252 in the C-terminal region of PA-X enhance the suppression of host gene expression [140,141]. In addition to the C-terminal tail, 22 amino acids in the N-terminus of PA-X are also important for nuclear localization and the shutoff activity of PA-X [142]. The N-terminus of PA-X is also found to be posttranslationally modified by an acetyltransferase, NatB [143]. The N-terminal E2 residue of PA-X and PA is acetylated, and the acetylation of this residue stimulates PA-X’s shutoff activity and promotes polymerase activity, although the mechanism is not well elucidated [143].

### 5.2. Role of PA-X in the Pathogenesis

As the shutoff of host translation is a viral evasion strategy, it is reasonable to predict that PA-X suppresses the expression of host innate immune responses. Indeed, PA-X frameshift mutant viruses induce higher IFNs and pro-inflammatory cytokines than their wild type counterparts [7,144,145]. A recent study showed that the PA-X-deficient virus elevates the expressions of Ifnb1 and Ifna4 in the mouse lungs in a MAVS-dependent manner [146]. However, the loss of PA-X increases IAV virulence in several animal models, including mice, chickens and ducks [7,144,145,147,148]. These studies suggest that the expression of PA-X decreases viral pathogenicity. There are a couple of explanations. One is that PA-X shuts off host innate immune responses, especially the expression of pro-inflammatory cytokines. The other one is that PA-X might affect viral replication. Two recent studies showed that PA-X inhibits viral polymerase activity [147,148]. However, how PA-X modulates viral RNA-dependent RNA polymerase (vRdRp) activity is not clear. It is also worthy of note that a study showed that the loss of PA-X in an avian H9N2 virus results in decreased virulence in mice [149]. In addition to PA-X, IAV has two other non-structural proteins, NS1 and PB-F2, which also antagonize host innate immunity, as discussed above. Therefore, the discrepancy might be due to the genetic variability of these three viral genes.

## 6. Other Non-Structural Proteins

### 6.1. PB1-N40

PB1-N40 was discovered as the third protein encoded by segment 2 of the IAV genome, which is the N-terminal truncate of PB1, the major product of segment 2 [8] (Figure 1). PB1-N40 does not form a complex with PA due to it missing the 39 N-terminal amino acids. Although PB1-N40 is not essential for IAV, the loss of this gene, in particular genetic backgrounds, is detrimental to virus replication [8]. Interestingly, the overexpression of PB1-N40 in a PB1-F2-deficient background also has a detrimental effect on virus growth in vitro and in vivo [150]. It might be context-dependent because PB1-F2 mutations affect PB1-N40 expression [8]. A proteomics study showed that PB1-N40 interacts with archain 1 (ARCN1), which is a component of the coat protein I (COPI) complex and required for IAV entry [151]. The knockdown of ARCN1 decreases H5N1 viral titer in chicken cells [152]. As PB1-N40 is a truncated form of PB1, it is not clear that this interaction is PB1-N40 specific. Moreover, how PB1-N40 participates in viral entry via ARCN1 needs further investigation. 

### 6.2. PA-N155 and PA-N182 

PA-N182 and PA-N155 are other two N-terminally truncated forms of PA, which are translated from the 11th and 13th in-frame AUG codons in the PA mRNA, respectively [9]. Although the start codons for PA-N182 and PA-N155 are highly conserved [9], whether natural isolates carry these proteins is not clear. These two truncate proteins have no effect on vRdRp activity; however, mutant viruses lacking these two proteins have lower replication activity in cells and lower pathogenicity in mice [9]. 

### 6.3. M42

The analysis of M transcripts found a fourth mRNA product (M4) in A/WSN/33 (H1N1) and a few other IAV strains [10]. This mRNA M4 encodes a new protein M42, which differs from M2 at the short N terminus but shares the same C-terminal 88 amino acids of M2 (Figure 1). Compared to M2, M42 occupies a lower proportion of the plasma membrane. Although altering the expression balance between M2 and M42 modulates virus pathogenicity, a virus only expressing M42 still causes significant disease, suggesting M42 could be a functional alternative to M2 [10].

### 6.4. NS3

A recent study showed that an A374G mutation in the NS1 gene leads to D125G(GAT→GGT) mutation in the coding [11]. More importantly, the A374G mutation activates a novel donor splice site, thus producing the novel viral transcript and protein termed NS3 [11]. NS3 is a truncated form of the NS1 protein, with an internal deletion of a motif comprising three antiparallel β-strands spanning codons 126 to 168 in NS1 (Figure 1). The GGT codon is found in 33 IAVs and is strongly associated with switching from avian to mammalian hosts [11]. The NS3 splice site and the resultant NS3 protein, but not the D125G substitution, are critical for the adaptive properties [11]. Future studies need to elucidate the underlying mechanisms and the differential roles of NS1 and NS3.

## 7. Conclusions and Future Directions

This review determines the important roles of NSPs in viral pathogenesis and host defense (Figure 2). The versatility of NSPs is further attributed to the diverse virulence and pathogenicity of IAV strains. Despite the increasing knowledge of NSP, the role of each NSP in different infection settings, such as viral strain and host tropism, is not well elucidated. Furthermore, much less is known for newly found NSPs. Recent advanced technologies now allow for systematically mapping IAV–host protein interaction networks, which has provided deep insights into IAV research. Several publications, including ours, have reported on a cohort of cellular factors interacting with IAV proteins by proteomics and yeast two-hybrid [36,99]. However, the cellular protein interaction network of most NSPs has yet to be explored. The discovery of novel binding host factors will delineate a more diverse set of cellular signaling pathways that are potentially associated with viral pathogenicity. Therefore, the further mapping of the interaction network of these viral proteins will provide unprecedented molecular insights into the role of NSPs. 

Mining additional NSPs from the IAV RNA genome is also an exciting study direction. Proteomics, ribosomal profiling and bioinformatic analysis have been applied in the virology field to dig out new genes or ORF. Bioinformatics searching has predicted a hypothetic protein in the NS segment, named NEG8 ORF. NEG8 ORF is translated from the genomic strand and is predicted to have a signal sequence and two transmembrane domains [153,154]. Moreover, a recent computational search, based on a ribosome scanning mechanism, identified 16 novel ORFs for all IAV genomes except NA and HA [155]. Although these ORFs are hypothetical, it would be interesting to verify these ORFs and their functions in the future. 

Since many NSPs antagonize the host innate immune defense, the deletion of some NSPs attenuates IAV virulence due to the more robust host immune response. As recombinant IAVs with NS1 deletion are attenuated, they have been used for the development of a live-attenuated influenza vaccine. For instance, an attenuated PR8 virus with a truncated NS1 protects aged mice from a lethal viral challenge [156]. The NS1-deficient influenza vaccine elicits a more robust immune response and has a better protective efficacy than an inactivated virus vaccine in aged mice [156,157]. Recently, a phase I/II clinical trial showed that an influenza virus vaccine lacking the NS1 is safe and induces high levels of antibodies [158]. As discussed above, other viral factors, such as PA-X and PB1-F2, also modulate host immune responses. In addition, a recent study generated temperature-sensitive IAVs by codon deletions of PA [159], which might be a potential candidate for live-attenuated vaccines. Future work will need to determine whether the attenuated IAVs with the deletion or mutation of single or multiple NSPs are suitable for vaccine development. 

Overall, these directions should improve our understanding of the role of NSPs in various IAV strains associated with different pathologies. Ultimately, the knowledge will help decipher the therapeutic targets and develop live-attenuated vaccines.
